# Anaemia prevalence, its determinants and profile of micronutrient status among rural school adolescent girls aged 14–19 years: a cross-sectional study in Nagpur district, Maharashtra, India

**DOI:** 10.1017/S1368980024002234

**Published:** 2024-11-11

**Authors:** Varsha S Dhurde, Archana B Patel, Lindsey M Locks, Patricia L Hibberd

**Affiliations:** 1 Lata Medical Research Foundation, Nagpur, Maharashtra, India; 2 Indira Gandhi Government Medical College, Nagpur, Maharashtra, India; 3 Adjunct Faculty Medical Research, Datta Meghe Institute of Medical Sciences, Wardha, Maharashtra, India; 4 Department of Health Sciences, Boston University, Boston, MA, USA; 5 Department of Global Health, Boston University School of Public Health, Boston, MA, USA; 6 Boston University School of Medicine, Boston, MA, USA

**Keywords:** Adolescent, Anaemia, Fe, Vitamin B_12_

## Abstract

**Objective:**

The objective of our study was to determine the prevalence of anaemia among 14–19 years school going girls, risk factors for it and profile of micronutrient status among rural girls from western state of India.

**Design:**

Using a cross-sectional design, we obtained information on socio-demography, menstruation, dietary habits, knowledge and daily consumption of the government recommended iron and folic acid (IFA) tablets, and anthropometry. Blood was collected to assess Hb, red blood cell indices, serumFe, folate and vitamin B_12_ levels.

**Settings:**

Nagpur district, Maharashtra, India.

**Participants:**

A total of 221 girls aged 14–19 years studying in twenty-four government institutes included.

**Results:**

57 % girls were anaemic, 84 % had deficiency of one or more micronutrients and 60 % were malnourished based on body mass index (BMI). The prevalence of Fe, vitamin B_12_ and folate deficiency was 37·7 %, 69·8 % and 1·4 %, respectively. Among anaemic girls, Fe and vitamin B_12_ deficiency was observed in 45·5 % and 67·5 %, respectively, *v*. among non-anaemic girls it was 27 % and 73 %, respectively. Fe deficiency was a predictor of anaemia and its severity. Girls residing in non-nuclear family were more likely to have anaemia. The consumption of daily non-vegetarian food and green leafy vegetables was 3 % and 3·6 %, respectively. Only 9 % consumed IFA tablets in the past 2 weeks.

**Conclusions:**

Anaemia is common in adolescent girls, particularly associated with Fe and vitamin B_12_ deficiency. There is need to reconsider the approach to prevention of anaemia in adolescent girls, particularly before they become pregnant.

Anaemia among young women has remained an arduous health challenge in India as 59 % of adolescent girls aged 15–19 years were anaemic in the National Family Health Survey (NFHS) conducted between 2019 and 2021^(1)^. The growth rate during adolescence is rapid and pubertal changes like onset of menarche occurring among girls during this phase expose them at greater risk to develop anaemia. Evidence suggests that even mild forms of anaemia can have deleterious impact on physical capacity and cognitive ability among adolescents^(2)^. Hence, detecting anaemia at its earlier stages or prior to the advancement to its clinical manifestation is crucial.

In view of the multifactorial aetiology of anaemia, it is equally imperative to identify its specific drivers. Systematic reviews of nationally representative surveys from India reported 37 % of anaemia to be associated with Fe deficiency among non-pregnant women of reproductive age^(3)^. A recent study from rural Haryana reports a prevalence of 29·6 % of Fe deficiency anaemia and 28·1 % of anaemia due to folate or vitamin B_12_ among adolescent girls^(4)^. Another study from South India observed that half of the women of child bearing age were deficient in folate and vitamin B_12_^(5)^. These variations first underscore the need to study region-specific pattern of micronutrient deficiencies. Second, it also emphasises the need to assess the challenges in the implementation of national Weekly Iron Folic acid Supplementation (WIFS) program. The program aims to provide free iron and folic acid (IFA) tablets (containing 100 mg of elemental Fe and 500 μg of folate) to adolescents on a weekly basis^(6)^, but issues of awareness and compliance may affect its effectiveness. In this study, our aim was to determine the prevalence of anaemia and its severity, the prevalence of Fe, folate and vitamin B_12_ deficiency and determinants of anaemia in rural school adolescent girls from Nagpur district.

## Methods

### Study design and settings

The study employed cross-sectional design. Data collection commenced from December 2019 to March 2020 in the rural areas of Nagpur district.

### Study participants

The study covered adolescent girls aged 14–19 year studying at educational institutes aided by the state government. The government aided educational institutes were selected as these are covered under national WIFS program.

### Sample size and sampling

Assuming the prevalence of anaemia among rural adolescent girls as 87 %^(7)^, precision of 5 %, 95 % CI and 20 % non-response rate the minimum required sample size was 214.

**Figure 1.**
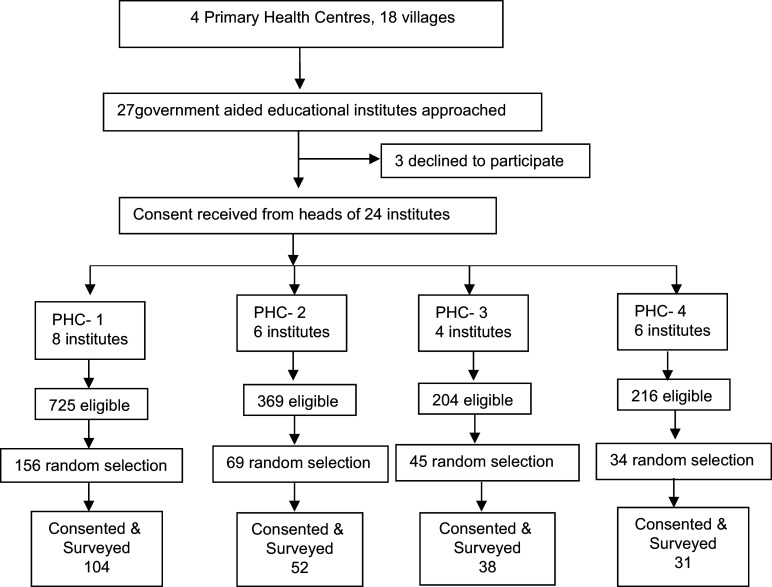
Sampling frame of the study.

A multistage purposive random sampling was used (Fig. 1). We selected the rural areas of Nagpur district from an ongoing Maternal Newborn Health Registry (MNHR) site. The MNHR has been collecting prospective data on maternal, fetal and neonatal outcome since 2009. Since its inception, the MNHR has registered more than 750 000 pregnant women and their babies in rural and semi-urban communities from various sites in Africa, Asia and Central America. Each site comprises between six and twenty-four distinct geographic locations (clusters, i.e. Primary Health Centres (PHC)). The rapport established with the public health department was helpful for the smooth conduct of the study. We first selected four PHCs that had villages with government aided educational institutes. The institutes that consented to participate provided a complete list of girls aged 14–19 years. We used population proportionate sampling of participants who were randomly chosen using online random number generator program.

### Inclusion criteria

We included those girls who were apparently healthy, had not received any blood transfusion in last three months, willing to provide consent for participating in the study and to withdraw blood for estimation.

### Data collection and measurements

Trained data collectors captured data from 225 girls on socio-demographics (total family size, type of family, parent’s education and occupation), menstruation (menarche status, age at menarche, number of days of bleeding due to menstruation per month), dietary habits (type of diet consumed (vegetarian or non-vegetarian), consumption frequency of green leafy vegetables, citrus fruits during the season and frequency of non-vegetarian food consumption- daily, weekly/monthly or never), knowledge and consumption of IFA tablets. Specific questions covered participants’ understanding of IFA supplementation, history of receiving IFA tablets, timing of the last receipt, reasons for any non-receipt, actual consumption of received tablets and reasons for any non-consumption in the past 14 d. Additionally, we inquired about a diagnosis of sickle cell disease, given its prevalence in the Nagpur district.

Data were captured electronically using Android tablets with a pre-tested questionnaire developed in Epi-collect 5. The data collectors were trained in anthropometric techniques. Weight (nearest 0·5 kg) of the subject was recorded using portable digital weighing scale (OMRON HN-286). Height (nearest 0·1 cm) was measured using portable stadiometer (Easy Care Stature Meter EC 1080) using standard procedures^(8)^. The household standard of living index was calculated using National Family Health Survey-2^(9)^.

A trained, experienced laboratory technician collected venous blood samples from the study subjects. Total 5 ml blood was drawn from each subject in two separate vacutainers – one with EDTA and other in plain. After blood draw, the samples were transferred to ice box within 30 min. The blood samples were sent for analysis to the Super Religare Laboratory on the same day for estimation. The Super Religare Laboratory is accredited by National Accreditation Board for Testing & Calibration. Vacutainers containing EDTA were used for estimation of Hb (Cyanmethemoglobin, Photometric, Horiba Micros ES 60). Plain vacutainers with blood were used for estimation of serum ferritin (electrochemiluminescence competitive immunoassay, Roche Cobas e411), serum folate, serum vitamin-B_12_ (electrochemiluminescence competitive immunoassay, Roche Cobas e411) and C-reactive protein (CRP) (Immunoturbidimetry, Siemens, Dade dimension Xpand plus).

### Data compilation and analysis

Data from the multiple android tablets were synced to the server and exported to Microsoft excel. After coding the file was exported into Epi-Info 7 and STATA®. National Family Health Survey-2 conducted in the year 1998–1999 provided an index reflecting economic well-being of a rural household based on number of variables such as household type, toilet facility, source of lighting in the house, type of fuel used for cooking, source of drinking water, presence of kitchen, ownership of house, agriculture land (irrigated/ non-irrigated), livestock and ownership of durable goods like mattress, cooker, chair, clock, cot, fan, cycle, radio, phone, refrigerator, television, scooter, bullock cart, crop thresher and tractor/car. Each variable is given a score, and sum of these scores is calculated. Scores of 0–14, 15–24 and 25–67 are defined as low, medium and high, respectively. The nutritional status of adolescent girls was evaluated using BMI (kg/m^2^). The BMI was derived by dividing the weight of girls (in kg) by the square of their height (in metres). The following BMI (kg/m^2^) categories were used to classify girls: <16 severely undernourished, 16–17 moderately undernourished, 17·1–18·4 mild undernourished, 18·5–22·9 normal and 23–24·9 overweight, >25 obese. BMI for age *Z* score (BAZ) and height for age *Z* score (HAZ) were calculated using WHO anthroplus software (2009). *Z*-score less than –2SD was regarded as wasted in case of BAZ and stunted in case of HAZ.

WHO cut-offs were used to define anaemia (Hb < 12 g/dl) and its grades (mild: 11·0–11·9 g/dl, moderate: 8·0–10·9 g/dl and severe: <8·0 g/dl)^(10)^ and deficiencies of Fe (serum ferritin <15 μg/l)^(11)^, folate (< 4 ng/ml) and vitamin B_12_ (< 203 pg/ml)^(12)^. Additionally, inflammation was assessed with CRP levels > 5 mg/l^(13)^.

Frequencies, prevalence statistic were derived using EPI-info7. Normality of all the continuous variables was assessed using Shapiro–Wilk’s test. For non-normally distributed continuous variables median (25^th^, 75^th^ percentile), and frequencies (%) for categorical variables were reported. Statistical analyses including univariate, bivariate and multinomial logistic regression analyses were performed using STATA®. For all statistical tests, a two-sided *P* value <0·05 was considered significant. Linear regression was performed to assess the association of factors with Hb level as the outcome variable. Multiple logistic regression was used to examine the factors associated with anaemia. Multinomial logistic regression was used to examine factors associated with the severity of anaemia among adolescent girls. We adjusted for standard of living index (high *v*. low -medium), type of family (nuclear *v*. non-nuclear), family size (<4 *v*. ≥ 4), type of diet consumed (vegetarian *v*. egg/ non-vegetarian), BMI for-age *z* score and height for age *z* score (normal *v*. BAz<–2sd, HAz <–2sd, respectively), serum ferritin and vitamin B_12_ (normal *v*. deficiency) status in our analysis.

## Results

**Figure 2.**
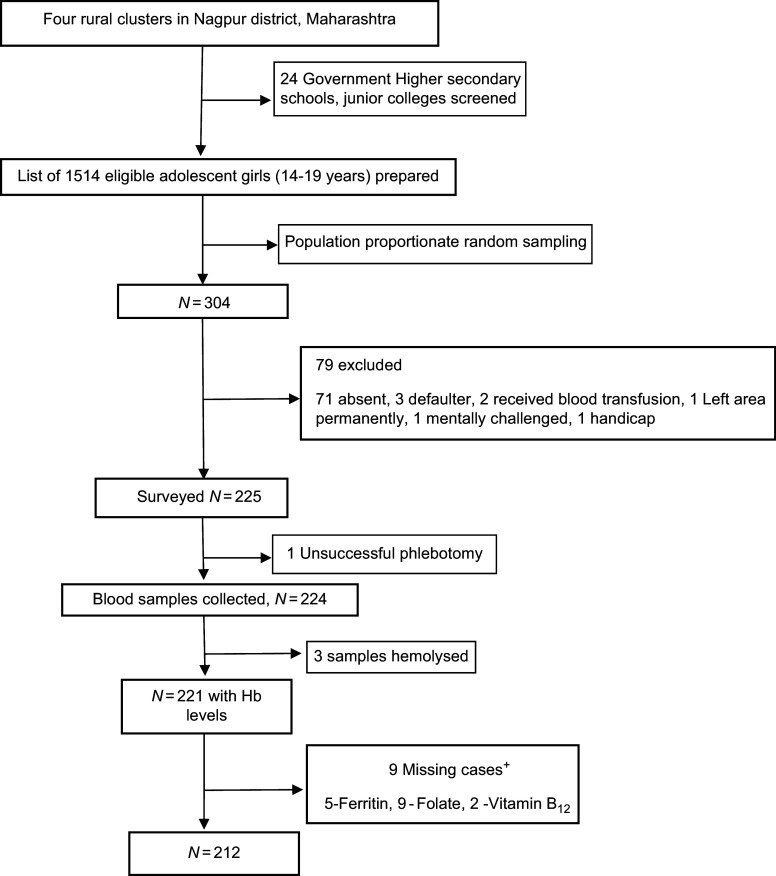
Analytical sample included all participants with required variables. Data for cases missing if the sample quantity was not sufficient or if the sample was spoilt. ^+^Not mutually exclusive.

There were 1514 eligible adolescent girls (14–19 year) from eighteen villages and twenty-seven educational institutes. Of these 304 girls were randomly selected from twenty-four consenting institutes of which 225 were eligible and consented. We were able to estimate the Hb in 221 and micronutrients in 212 girls (Fig. 2).

### Socio-demographic characteristics and menstrual history of the study population (n 221)

The majority (84 %) of the girls was aged 14–17 -years (Table 1). The standard of living index as described in National Family Health Survey-2 was calculated. Majority of these girls belonged to high standard of living index. More than half of the girls (58 %) belonged to nuclear family, and most of the girls (93 %) had four or more members in the family. Above 80 % of the girls’ parents were literate, and the mothers of 68 % of the girls were working. Only 2 % of the girls had not attained their menarche. The median (25^th^, 75^th^ percentile) age at menarche was 14 (13, 15) years. Of the girls who had attained their menarche, 72 % reported to have four or more days of bleeding during menstruation.

**Table 1.** Socio-demography, menstrual history, sickle cell disease status and knowledge of iron folic acid (IFA) tablets, duration of receipt and consumption of IFA tablets of the population

Variables		Frequency (*n*)	Percentage (%)
Age group (years)	14–17	185	84·0
	18–19	36	16·0
Standard of living index (SLI)	(0–14) Low	2	1·0
	(15–24) Medium	19	9·0
	(25–67) High	200	90·0
Family type	Nuclear	129	58·0
	Non-nuclear	92	42·0
Family size	<4	16	7·0
	≥ 4	205	93·0
Mothers’ education	Illiterate	23	10·0
	Literate	188	85·0
	Do not know	10	5·0
Mothers’ occupation	Housewife	64	29·0
	Working	151	68·0
	Do not know	6	3·0
Fathers’ education	Illiterate	17	8·0
	Literate	186	84·0
	Do not know	18	8·0
Fathers’ occupation	Non-working	2	1·0
	Working	205	93·0
	Do not know	14	6·0
Menarche status	Attained	216	98·0
	Not attained	5	2·0
Number of days of bleeding during menstruation	<4	61	28·0
	>=4	155	72·0
Sickle cell disease status	Yes	5	2·0
	No	183	83·0
	Do not know	33	15·0
Identification and having previously received IFA tablets	Yes	208	94·0
	No	13	6·0
Knowledge regarding use of IFA tablets	Having knowledge of anaemia	123	56·0
	No knowledge of anaemia	98	44·0
Consumption of IFA tablets ever received	Yes	194	93·0
	No/ Do not know	14	7·0
Period of last receipt of IFA tablets	≤ 2 months	80	38·0
	>2 and ≤ 4 month	31	15·0
	> 4 months	97	47·0
Consumption of IFA tablets in last 14 d	Yes	20	9·0
	No	201	91·0

### Sickle cell disease status

Five girls of the 221 had history of being diagnosed with sickle cell disease.

### Knowledge, duration of receipt and consumption of iron and folic acid tablets

Although most (94%) of the girls could identify IFA tablets (packets of tablets shown to them) having received in the past, only 56% reported understanding its purpose to prevent anaemia (Table 1). Almost half (47%) of the girls reported to have received IFA tablets more than 4 months ago and the girls those who had received IFA tablets, majority had consumed the tablets but only 9 % of the girls reported to have consumed the tablets in the past 2 weeks.

### Dietary habits and nutritional status

Almost one-third of the study population was vegetarian (i.e. did not consume meat), but their daily diet was devoid of green leafy vegetables (Table 2). Daily citrus fruit consumption was found in one-fifth of the girls, and daily consumption of non-vegetarian food was found in only 3 % of the girls despite 72 % of the girls reported that they consumed eggs/non-vegetarian food. Almost 50 % of the girls were undernourished (BMI < 18·5 kg/m^2^) and of this, half were in the severe to moderate category (BMI < 17 kg/m^2^). The BAZ and the HAZ scores (calculated till 19 years of age) showed that 11 % were wasted and a third was stunted.

**Table 2.** Dietary habits and nutritional status of population

Variables	Frequency (*n*)	Percentage (%)
Type of diet consumed		
Vegetarian	63	28·0
Consume eggs/ non-vegetarian	158	72·0
Frequency of green leafy vegetables consumption		
Daily	8	3·6
Weekly/ monthly	212	96
Never	1	0·4
Frequency of citrus fruit consumption		
Daily	43	20·0
Weekly/monthly	169	76·0
Never	9	4·0
Frequency of eggs/ non-vegetarian consumption		
Daily	4	3·0
Weekly/monthly	154	97·0
BMI (kg/m^2^)		
< 16 – Severe	22	10·0
16–17 -Moderate	24	11·0
17·1– 18·4 – Mild	59	27·0
18·5–22·9 – Normal	89	40·0
23–24·9 – Overweight	12	5·0
>25 – Obese	15	7·0
BMI-for-age *Z*-score (BAZ)*		
BAZ <–2 s d	24	11·0
Normal	190	89·0
Height-for-age *Z* score (HAZ)*		
HAZ <–2 sd	71	33·0
Normal	143	67·0

* : *n* 214 Data for seven cases could not be calculated as their age was >228 months (19 years).

### Anaemia prevalence

**Figure 3.**
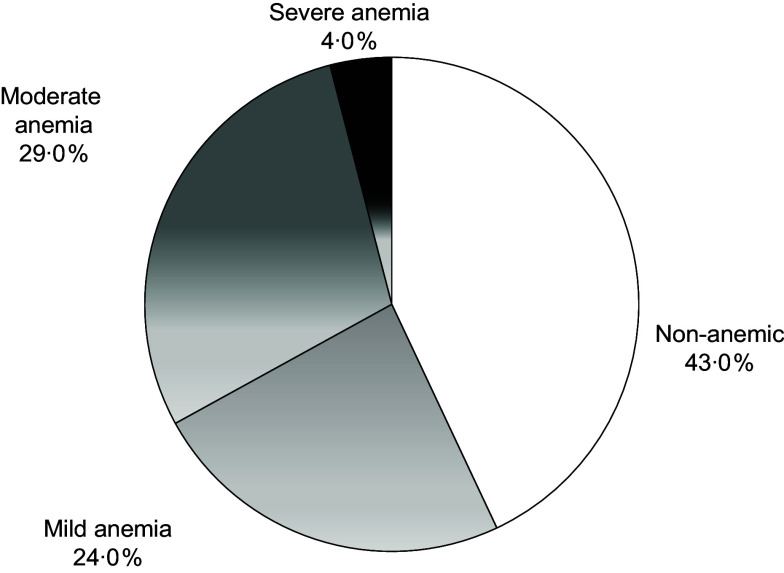
Prevalence and severity of anaemia in the study population (*n* 221).

Anaemia was observed in 57 % (95 % CI = 50·8, 63·8) of the girls. Mild, moderate and severe anaemia was present in 24 %, 29 % and 4 %, respectively (Fig. 3).

### Micronutrient status in anaemic and non-anaemic girls (n 212)

**Figure 4.**
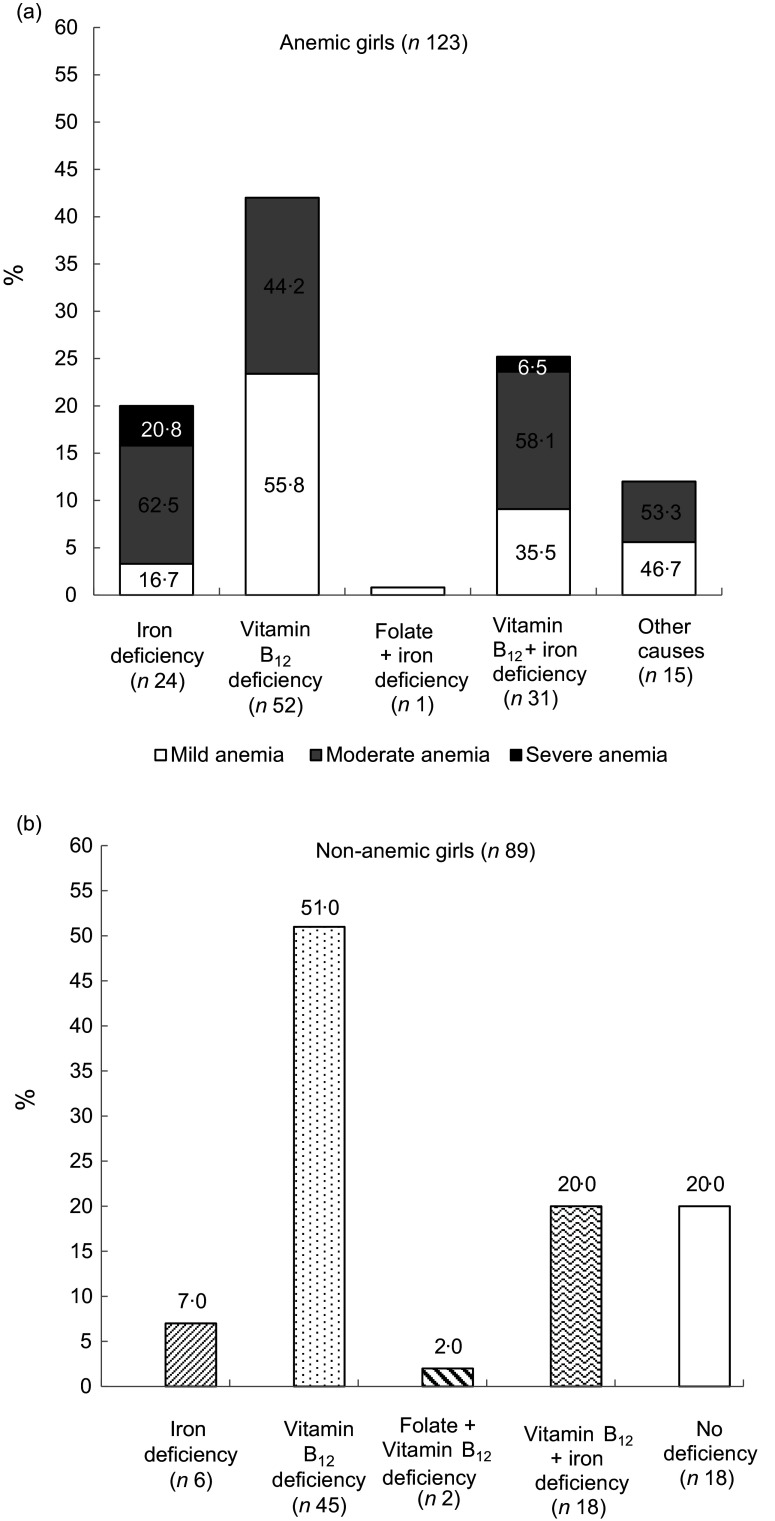
Status of micro-nutrients in study population by anaemia status.

Deficiency of any micronutrient was observed in 88 % (108/123) of anaemic and 80 % (71/89) of the non-anaemic girls. The presence of Fe, vitamin B_12_ and folate deficiency was observed in 37·7 %, 69·8 % and 1·4 % girls, respectively, regardless of their anaemic status (Table 3). In anaemic girls, the presence of Fe, vitamin B_12_ and folate deficiency was 45·5 %, 67·5 % and 0·8 %. The distribution of mild, moderate and severe anaemia in those with Fe deficiency was 28·5 %, 59 % and 12·5 %. The distribution of mild, moderate and severe anaemia in those with vitamin B_12_ deficiency was 48·2 %, 49·4 % and 2·4 %, respectively. Only one girl had folate deficiency and anaemia was mild. Anaemia due to other causes was observed in 15 (12 %) girls (Fig. 4(a)).

**Table 3.** Distribution of girls by micro-nutrient and anaemia status (*n* 212)

Micro-nutrient status			Non-anaemic n (%) 89 (42·0)		Total anaemic n(%) 123 (58·0)		Anaemia grades n (%)					
							Mild 52 (42·3)		Moderate 64 (52)		Severe 7 (5·7)	
	*n*	%	*n*	%	*n*	%	*n*	%	*n*	%	*n*	%
Fe*	Deficiency 80	37·7	24	27·0	56	45·5	16	28·5	33	59·0	7	12·5
	Normal 132	62·3	65	73·0	67	54·5	36	53·7	31	46·3	0·0	
Vitamin- B_12_ †	Deficiency 148	69·8	65	73·0	83	67·5	40	48·2	41	49·4	2	2·4
	Normal 64	30·2	24	27·0	40	32·5	12	30·0	23	57·5	5	12·5
Folate‡	Deficiency 3	1·4	2	2·3	1	0·81	1	100	0·0		0·0	
	Normal 209	98·6	87	97·7	122	99·2	51	41·8	64	52·5	7	5·7

* : This may include vitamin B_12_ or folate deficiency also.

† : This may include Fe or folate deficiency.

‡ : This may include Fe or vitamin B_12_ deficiency.

Exclusive Fe deficiency was observed in 20 % of anaemic girls, whereas exclusive vitamin B_12_ deficiency was found in 42 % of anaemic girls. Among anaemic girls with exclusive Fe deficiency (*n* 24), the distribution of mild, moderate and severe anaemia was 16·7 %, 62·5 % and 20·8 %, respectively (Fig. 4(a)). Girls having exclusive vitamin B_12_ deficiency had only mild (55·8 %) and moderate (44·2 %) forms of anaemia. Girls who did not have any deficiency (*n* 15) but anaemia due to some other reasons had mild (46·7 %) and moderate (53·3 %) anaemia.

Among non-anaemic girls, forty-five girls (51 %) had only vitamin B_12_ deficiency, six (7 %) had only Fe deficiency and concomitant deficiency was observed in eighteen (20 %) girls (Fig. 4(b)). Folate deficiency existed in combination with vitamin B_12_ deficiency among only 2 (2 %) girls.

The multivariate analysis for anaemia showed that girls belonging to non-nuclear family tended to be at a higher risk for anaemia (adjusted OR = 1·7, CI = 0·98 –3·2; *P* = 0·057). However, the strongest predictor of anaemia was Fe deficiency (adjusted OR = 2·38, CI = 1·29, 4·4; *P* = 0·005) (Table 4). The multinomial logistic regression showed that Fe deficiency was the only predictor of moderate-severe form of anaemia (adjusted OR = 3·58, CI = 1·79, 7·2) (Table 5).

**Table 4.** Multivariate logistic regression for anaemia (*n* 209)†

Characteristics	*N*	Anaemia (*n* 119)		No anaemia (*n* 90)		COR	95 % CI	*P*- value	AOR	95 % CI	*P*-value
		*n*	%	*n*	%						
Standard of living index (SLI)											
High SLI	189	110	58·2	79	41·8	1·7	0·67, 4·3	0·37	1·4	0·51, 3·6	0·53
Low-medium SLI	20	9	45·0	11	55·0						
Family type											
Non-nuclear	88	58	66·0	30	34·0	1·9	1·1, 3·4	0·03*	1·7	0·98, 3·2	0·057
Nuclear	121	61	50·4	60	49·6						
Family size											
< 4	14	7	50·0	7	50·0	1·34	0·45, 3·9	0·79	0·98	0·31, 3·1	0·97
≥4	195	112	57·4	83	42·6						
Type of diet											
Vegetarian	60	33	55·0	27	45·0	1·1	0·61, 2·0	0·83	1·2	0·6, 2·2	0·65
Eggs/non-vegetarian	149	86	57·7	63	42·3						
BMI-for-age *Z*-score (BAZ)											
Normal	186	105	56·5	81	43·5	1·2	0·49, 2·91	0·85	1·1	0·43, 2·9	0·77
BAZ < –2 s d	23	14	60·9	9	39·1						
Height-for-age *Z* score (HAZ)											
Normal	138	75	54·3	63	45·7	1·36	0·76, 2·45	0·36	1·5	0·79, 2·8	0·21
HAZ < –2 s d	71	44	62·0	27	38·0						
Serum ferritin											
Normal	129	63	48·8	66	51·2	2·44	1·35, 4·4	0·004**	2·38	1·29, 4·4	0·005**
Fe deficient	80	56	70·0	24	30·0						
Serum vitamin B_12_											
Normal	63	38	60·3	25	39·7	0·82	0·44, 1·5	0·62	0·93	0·49, 1·76	0·83
B_12_ deficient	146	81	55·5	65	44·5						

† *n* 209, data for three participants for HAZ and BAZ could not be calculated as their age was greater than 228 months (19 years); AOR, adjusted OR; COR, crude OR; *: *P* < 0.05, **: *P* < 0.01.

**Table 5.** Multinomial logistic regression analysis results on anaemia severity among adolescent girls by socio-demographic and nutritional characteristics

Characteristics	*N*	Anaemia status by Hb levels n (%)†									
		No anaemia –90 (43·1) (Ref)		Mild anaemia-50 (23·9)				Moderate-severe anaemia-69 (33)			
		*n*	%	*n*	%	AOR	95 % CI	*n*	%	AOR	95 % CI
Standard of living index (SLI)											
High SLI	189	79	41·8	46	24·3	0·66	0·19, 2·3	64	33·9	0·78	0·24, 2·6
Low-medium SLI	20	11	55·0	4	20·0			5	25		
Family type											
Non-nuclear	88	30	34·1	25	28·4	1·91	0·92, 3·9	33	37·5	1·69	0·85, 3·4
Nuclear	121	60	49·6	25	20·7			36	29·7		
Family size											
<4	14	7	50·0	3	21·4	1·01	0·24, 4·4	4	28·6	1·03	0·26, 4·1
≥4	195	83	42·6	47	24·1			65	33·3		
Type of diet											
Vegetarian	60	27	45·0	14	23·3	0·83	0·38, 1·8	19	31·7	0·87	0·42, 1·8
Eggs/non veg	149	63	42·3	36	24·2			50	33·5		
BMI-for-age *Z*-score (BAZ)											
Normal	186	81	43·5	45	24·2	1·04	0·32, 3·4	60	32·3	1·2	0·42, 3·5
BAZ < –2 s d	23	9	39·1	5	21·8			9	39·1		
Height-for-age *Z* score (HAZ)											
Normal	138	63	45·7	29	21	1·7	0·79, 3·6	46	33·3	1·33	0·64, 2·7
HAZ < –2 s d	71	27	38·0	21	29·6			23	32·4		
Serum ferritin											
Normal	129	66	51·1	34	26·4	1·29	0·59, 2·8	29	22·5	3·58**	1·79, 7·2
Fe deficient	80	24	30·0	16	20·0			40	50		
Serum vitamin B_12_											
Normal	63	25	39·7	12	19·0	1·26	0·55, 2·9	26	41·3	0·76	0·37, 1·6
B_12_ deficient	146	65	44·5	38	26·0			43	29·5		

† *n* 209 data for three participants for HAZ and BAZ could not be calculated as their age was >228 months (19 years). AOR: adjusted OR; *: *P* < 0.05, **: *P* < 0.01.

## Discussion

We found 57 % of adolescent rural girls in Nagpur district were anaemic. Our finding is in agreement with current national anaemia estimate^(1)^ and the reported studies^(14)^ from rural Maharashtra conducted 15 years ago. This indicates that anaemia among adolescent girls remains a major public health problem^(15)^ in this region.

In our study, we observed overall 38 % of the girls had Fe deficiency, which is moderate public health problem^(11)^. Few other studies conducted in rural communities from Maharashtra have reported 41 % Fe deficiency (ferritin < 15 μg/l) among 12–16 year adolescents from boarding school from Ahmednagar district^(16)^, which is consistent with our result. A study from Wardha district reported 67 % Fe deficiency (ferritin < 12 μg/l), 50 % Fe deficiency anaemia among 12- to 15-year-old girl students^(17)^. We observed about 46 % Fe deficiency anaemia (Table 3), which is in consonance with this study and the generally postulated opinion that Fe deficiency accounts for almost half of anaemia among children, adolescents and women of reproductive age^(18)^. Almost one-third of the non-anaemic girls in our study had Fe deficiency. Studies reporting Fe deficiencies among non-anaemics are few,^(4,17)^ and the prevalence ranges between 11 and 17 %. The variations in prevalence could be due to variations in regional dietary patterns, wealth index, rates of infections, compliance to WIFS program and also difference in the cut-offs of serum ferritin used to define Fe deficiency.

The median (25^th^, 75^th^ percentile) levels of serum ferritin among anaemic Fe deficient girls in our study was 6·4 μg/l (4·6, 8·4) and 10·3 μg/l (6·8, 13·1) among non-anaemic Fe deficiency, which is also known as stage I (storage depletion)/stage II (mild functional deficiency stage) of Fe deficiency^(18)^. Our result thus presents functional lower reference limit of serum ferritin, which is associated with transition from deficiency into anaemic stage. Thus, our study indicates threshold levels of serum ferritin (10·3 μg/l) below which Fe-deficient erythropoiesis occurs manifesting into anaemia^(19)^.

Fe deficiency was not only a predictor of anaemia but also of its severity. Among several socio-demographic factors, it was found that girls residing in non-nuclear family had higher prevalence (66 %) of anaemia (*P* value= 0·057) as compared with girls residing into nuclear family (50·4 %) (Table 4). Similar findings have been reported by other studies^(20)^. This could be because the foods like lentils, nuts and non-vegetarian foods are rich in proteins but are expensive. So, in non-nuclear family set up, it is less likely to be consumed which could be cause of anaemia among girls. Second, it is common to find that males in patriarchal Indian rural families may have significant advantages in terms of quality and quantity of food consumed. Although majority (72 %) of the girls belonged to families that consumed non-vegetarian food, the frequency of its daily consumption among the girls was observed in only 3 %. Similarly, daily green leafy vegetable consumption was observed in only 3·6 %. This indicates that their diet was predominantly of cereals and lentils. This could be attributed to the finding that in our study one-third (33 %) of the girls had chronic malnutrition (HAZ < –2SD), and the percentage of girls having acute malnutrition (BMI < 18·5) was almost 50 %. Integrating Fe-fortified cereals like rice into school mid-day meals can be an effective strategy for improving Fe status and anaemia in the region where Fe deficiency is prevalent^(21)^.

Serum ferritin concentrations are elevated during inflammation. CRP and a-1-acid glycoprotein are the two commonly used markers to detect underlying inflammation^(11)^. We have estimated only CRP and found that only four girls had elevated (> 5 mg/l) CRP. Excluding these girls, the prevalence of Fe deficiency (80/208, i.e. 38·5 %) did not differ. The observed Fe deficiency in our study could be attributed to failure of regular consumption of IFA tablets as nearly half the girls had not received IFA tablet in the last 4 months and only 9 % had received it in the 2 weeks prior to the interview (Table 1). A recent study has shown low coverage in receiving IFA supplements among adolescents in preceding year of the survey conducted during 2015–2016^(22)^. Inadequate supply of IFA tablets has remained one of the bottlenecks in the WIFS implementation and the extent of which vary widely across the districts^(23)^.

A more comprehensive analysis of adolescent girls’ knowledge about IFA tablets, the obstacles to their consumption and the supply issues will be covered in separate article.

Majority of the girls (69·8 %) in our study had deficiency of vitamin B_12_. The predominance of vitamin B_12_ deficiency among adolescents has also been reported from north and eastern India^(24,25)^ with the prevalence in the range of 50–90 %. The studies examining vitamin B_12_ status among adolescents from central India are scarce. In our study, only 3 % girls consumed non-vegetarian foods daily which could be the cause of high prevalence of vitamin B_12_ deficiency among them. Out of total non-anaemic girls, 51 % were vitamin B_12_ deficient (Fig. 4(b)). Very few studies^(4)^ in India report vitamin B_12_ status among non-anaemic girls. The high rate of vitamin B_12_ deficiency without haematologic changes may go unnoticed^(26)^. Deficiency of vitamin B_12_ may affect reproduction and cause frequent abortions^(12)^. Hence, it needs attention earlier during adolescence as the symptoms of deficiency can be reversed with early diagnosis and treatment^(27)^. To find out the extent of deficiency among individuals to help prioritise and plan future interventions, screening like National Health and Nutrition Examination Survey is needed.

We found only 1·4 % folate deficiency in our study population. This may be due to adequacy of dietary folate. Only 3·6 % girls reported to consume green leafy vegetables daily. This could be perhaps because the question was framed to ask consumption of specifically green leafy vegetables as a vegetable preparation. The respondents may have failed to report or overlooked addition of spinach or other green leafy vegetables in lentils which is staple food in this region. The study from Himachal Pradesh^(24)^ also did not find folate deficiency among adolescent boys and girls. We observed 12 % of the adolescent girls had anaemia without Fe, folate or vitamin B_12_ deficiency. Anaemia may also be due to multiple other micronutrients deficiency and estimations of these were beyond the scope of our study. Similarly, possibility of anaemia due to non-nutritional factors like bleeding due to menstruation, worm infestations, peptic ulcers, gastritis or other haemoglobinopathies could not be ruled out.

The major strengths of our study are that it provided regional estimates of anaemia and common concomitant micronutrient deficiencies in rural adolescent girl population that was being served by the national WIFS program. We not only used high-quality standardised laboratory methods to estimate hematologic parameters and micronutrient levels in the blood but also assessed their demographics, dietary habits and caveats in the regional implementation of the WIFS program. The current findings are specific to the study area, characterised by high malnutrition rates and may not be applicable to different settings. Further, the limitations are that we did not assess dietary intake of micronutrients, which would have provided better insights into dietary patterns among these girls. We selected rural clusters with education institutes where WIFS program was being implemented so the generalisability of this study to other rural regions where educational institutes are not present will be limited.

## Conclusions

Our study fills a gap on micronutrient deficiencies among rural adolescent girls from central India. Majority of the girls from our study were anaemic, 84 % had deficiency of one or more micronutrients. While vitamin B_12_ deficiency remained the predominant deficiency, it was Fe deficiency that was predictive of anaemia and its severity. We conclude that Fe deficiency remains a moderate public health problem^(11)^. Our study highlights the need of strengthening the WIFS program and underscores the need for screening to unveil micronutrient deficiencies to inform preventive public health action.

## Data Availability

The data used for this study are available from corresponding author on reasonable request.
